# Changes in Temperature Sensitivity and Activation Energy of Soil Organic Matter Decomposition in Different Qinghai-Tibet Plateau Grasslands

**DOI:** 10.1371/journal.pone.0132795

**Published:** 2015-07-15

**Authors:** Jie Li, Nianpeng He, Xuehong Wei, Yang Gao, Yao Zuo

**Affiliations:** 1 Key Laboratory of Ecosystem Network Observation and Modeling, Institute of Geographic Sciences and Natural Resources Research, Chinese Academy of Sciences, Beijing, 100101, China; 2 Institute of Grassland Science, Northeast Normal University, Changchun, 130024, China; 3 Key Laboratory of Vegetation Ecology, Ministry of Education, Changchun, 130024, China; 4 Agricultural and Animal Husbandry College of Tibet University, Linzhi, 860000, China; Fudan University, CHINA

## Abstract

Qinghai-Tibet Plateau grasslands are unique geographical regions and store substantial soil organic matter (SOM) in the soil surface, which make them very sensitive to global climate change. Here, we focused on three main grassland types (alpine meadow, steppe, and desert) and conducted a soil incubation experiment at five different temperatures (5, 10, 15, 20, and 25°C) to investigate SOM decomposition rates (*R*), temperature sensitivity (*Q*_10_), and activation energy (*E*_a_). The results showed that grassland type and incubation temperature had significant impact on *R* (*P* < 0.001), and the values of *R* were exponential correlated with incubation temperature in three alpine grasslands. At the same temperature, *R* was in the following order: alpine meadow > alpinesteppe > alpine desert. The *Q*_10_ values differed significantly among different grasslands, and the overall trends were as follows: alpine meadow (1.56 ± 0.09) < alpine steppe (1.88 ± 0.23) < alpine desert (2.39 ± 0.32). Moreover, the *E*_a_ values differed significantly across different grassland types (*P* < 0.001) and increased with increasing incubation time. The exponential negative correlations between *E*_a_ and *R* at 20°C across all grassland types (all *P*s < 0.001) indicated that the substrate-quality temperature hypothesis is applicable to the alpine grasslands. Our findings provide new insights for understanding the responses of SOM decomposition and storage to warming scenarios in this Plateau.

## Introduction

The decomposition of soil organic matter (SOM) is an important flux of CO_2_ to the atmosphere [1]. Temperature is an important factor regulating the decomposition of SOM. In practice, temperature sensitivity (*Q*_10_) has been widely used to dipict the responses of SOM decomposition rate (*R*) to temperature changes, where *Q*_10_ was used as a factor to indicate the increasing *R* with a 10°C increase in temperature [1,2]. Since *Q*_10_ values for various terrestrial ecosystems are not yet known, scientists set these values as a constant (2.0) in exponential equations to simulate the effect of changing temperature on *R*, such as in the terrestrial ecosystem model (TEM) [3–5]; this results in an apparent uncertainty of estimation. In some studies, *Q*_10_ values showed greater variation (1.2–5.9) in time and space both in natural ecosystems and in incubation experiments [6,7]. The variability of *Q*_10_ across different terrestrial ecosystems needs to be extensively evaluated and incorporated into biogeochemical models to improve the estimation.

In the past decades, the effects of environmental factors (such as temperature and moisture) on *R* and the underlying mechanisms have been determined for various ecosystems. However, fewer studies have focused on the mechanism controlling respiratory substrates [8]. One difficulty is to understand the microbial mechanisms, which are influenced by temperature, enzymatic reactions (extra and intracellular), diffusion of substrates, and microbial substrate utilization efficiency [9]. The temperature response of reactions at all scales of life can be determined by the activation energy (*E*_a_) to some extent [10]. The activation energy theory, which was developed from the temperature sensitivity theory, indicated that the temperature sensitivity of SOM decomposition depends on SOM quality, namely, the molecular weight, molecular structure complexity, and chemical bond stability. The activation energy theory therefore provide a framework for predicting the temperature sensitivity of SOM decomposition under different soil quality or conditions. By using the activation energy theory and the classic Arrhenius equation, scientists proposed the substrate-quality temperature hypothesis [11]. This theory suggests that complex organic compounds generally have lower *R* and higher *E*_a_; with increasing in molecular weight and molecular structure of SOM, the higher energy required to initiatea reaction among these organic compounds, thus the temperature sensitivity increased correspondingly [12]. This hypothesis has been verified in North America [10] and Europe [13]. However, whether this hypothesis is applicable to the unique grasslands of the Qinghai-Tibet Plateau has not yet been experimentally confirmed.

The Qinghai-Tibet Plateau is unique ecological module [14] due to its high altitude, and is considered as one of the world’s most sensitive areas to global climate change [15]. The plateau provide a unique opportunity to explore the feedback of SOM decomposition to climate change [16]. The grassland area of this plateau is approximately 1.4 × 10^8^ hm^2^, and there is apparent accumulation of SOM on the soil surface (0–10 cm soil layer stored about 30% soil organic carbon [SOC]) [17].

The global mean annual temperature increased by 0.74°C during the last century and has been estimated to increase by 1.1–6.4°C by 2100 [18]. Under warming scenarios, the intrinsic SOM storage in the soil surface of the Qinghai-Tibet Plateau grasslands might accelerate decomposition, resulting in substantial loss of SOC. Therefore, determining the temperature sensitivity of SOM decomposition in the grasslands of the Qinghai-Tibet Plateau is essential to accurately predict its response to future climate warming [19].

In this study, we focused on three main grassland types (alpine meadow, steppe, and desert) and conducted soil incubation experiments at five different temperatures (5, 10, 15, 20, and 25°C), to investigate *R*, *Q*_10_, and *E*_*a*_. The main objectives were: (1) to investigate the differences in *R*, *Q*_10_, and *E*_*a*_ among different alpine grasslands, and (2) to verify whether the substrate-quality temperature hypothesis is applicable to the Qinghai-Tibet alpine grasslands.

## Materials and Methods

### Study sites

**Fig 1:**
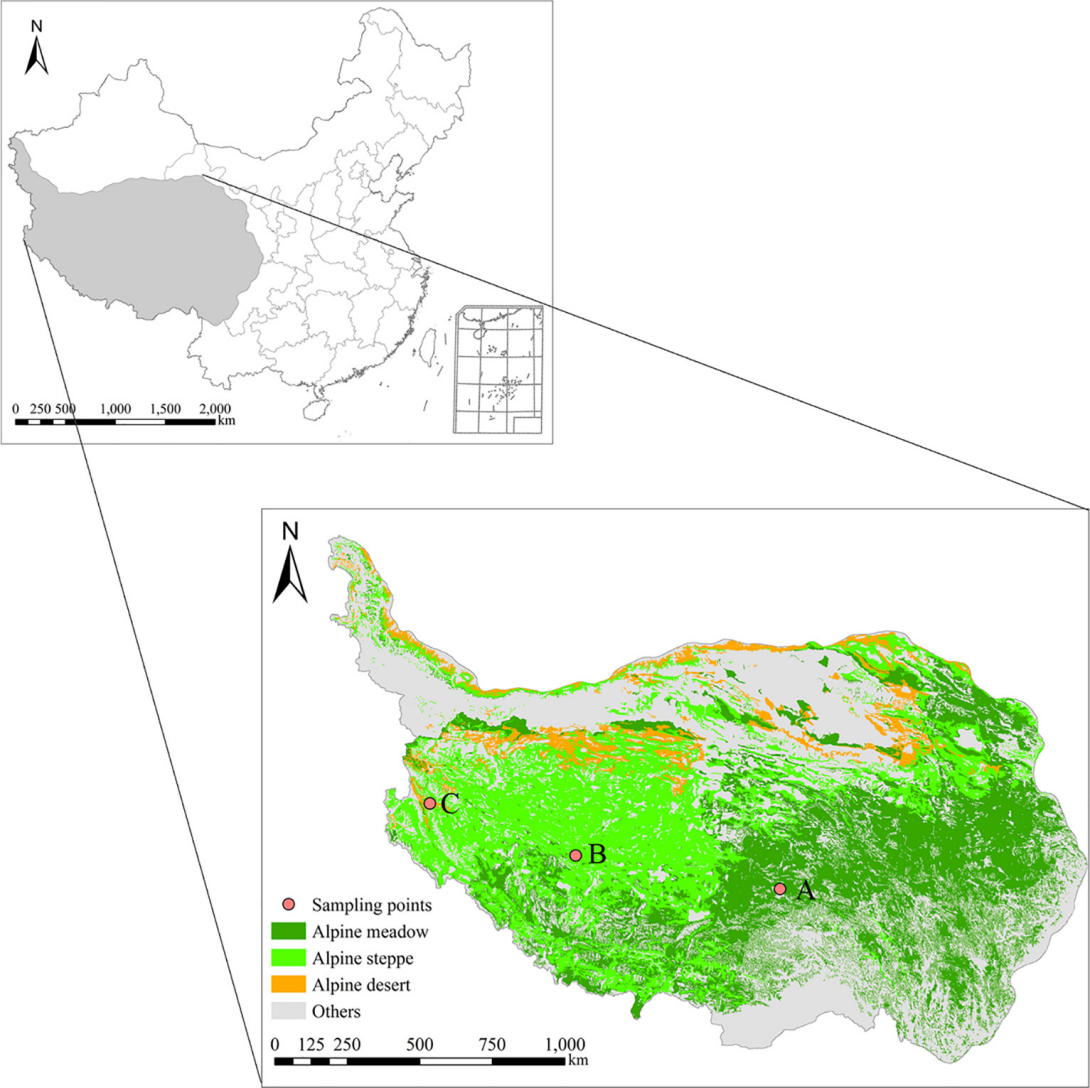
Location of the experimental plots and main grassland types in the Qinghai-Tibet Plateau.

The Qinghai-Tibet Plateau is the highest plateau in the world, with an average elevation of 4000 m (26°10′–39°30′N, 73°20′–104°20′E; Fig 1). The plateau has continental monsoon climate that varies across different regions [20]. The experimental plots were selected from three main grassland types distributed widely in the Qinghai-Tibet Plateau (Fig 1), these were designated as alpine meadow (A), alpine steppe (B), and alpine desert (C).

The experimental plots of alpine meadow located on the eastern Qinghai-Tibet Plateau in Suo County, Nagqu City. The area is characterized by moist grass. Soil is subalpine shrub meadow soil. The plant community at the experimental plots is dominated by *Kobresia humilis*, *Leontopodium nanum*, and *Thalictrum alpinum* (seeing Table 1 for detail). The experimental plots of alpine steppeare located in Nima County of midwest Qinghai-Tibet Plateau. This region belongs to the alpine grass semiarid area and is characterized by low temperatures and less rainfall. Soil belong to alpine steppe soil, which is relatively shallow with fine grain structure and cataclastic rocks. The dominant vegetation consists of *Carex moorcroftii* and *L*. *alpinum*. The experimental plots of alpine desert located in Gaer County of west Qinghai-Tibet Plateau. The area belongs to the alpine grassland drought area in northern Qinghai-Tibet Plateau. Soils belong to alpine cold desert soil. The grassland vegetation is dominated by *Ajania fruticulosa* and *Stipa glareosa* [20, 21].

**Table 1:** Selected characteristics of the experimental plots.

Grassland types	Location	MAT^†^(°C)	MAP (mm)	Altitude(m)	Dominant species	Coverage (%)	Biomass (g m^–2^)
Alpine meadow	31°52′15″N93°30′52″E	3.1	572.9	4535	*Kobresia humilis Leontopodium nanum*	89 ± 7^a^ ^‡^	147.01 ± 12.46^a^
Alpine steppe	31°55′15″N 85°50’35″E	–1.9	150.0	4907	*Carex moorcroftii Leontopodium alpinum*	20 ± 2^b^	51.23 ± 3.43^b^
Alpine desert	32°29′49″N 80°08′6″E	0.1	73.4	4294	*Ajania fruticulosa Stipa glareosa*	8 ± 2^c^	14.36 ± 1.89^c^

^**†**^ MAT, mean annual temperature; MAP, mean annual precipitation

^‡^ The same small letters in different grassland type indicated no significant difference at *P* = 0.05 level.

### Field sampling

Field sampling was conducted in August 2013. In each plot, eight sampling quadrats (0.5 m × 0.5 m) were established at 10 m intervals along a random transect.

The community structure was investigated in 1, 3, 5, and 7 quadrats, and aboveground biomass was investigated in 2, 4, 6, and 8 quadrats. Subsequently, surface litter was removed and soil samples (about 5 kg) were randomly collected using a soil sampler (10 cm in diameter) from the surface soil (0–10 cm) from 1, 3, 5, and 7 quadrats. Samples from the same depth were pooled, packed in polyethylene bags, immediately stored in a portable refrigerator (4°C), and transported to the laboratory. The samples were passed through a 2 mm sieve, and then all the visible plant materials were manually removed from the sieved soils. Approximately 100 g of each soil sample was air-dried for analysis of soil properties (e.g., C, N, and pH). The remaining soil was stored at 4°C.

The plots were public lands in China and no specific permissions were required for these locations/activities to conduct ecological researches. Moreover, this field study did not include any endangered or protected species.

### Laboratory incubation and analysis

#### Chemical analyses

The concentration of SOC (%) was determined using by using the loss-on-ignition method with K_2_Cr_2_O_7_–H_2_SO_4_ [22]. Microbial biomass carbon (μg g^–1^) was determined using the chloroform fumigation-extraction method [23]. The N concentration of soil (%) was measured using a modified Kjeldahl wet-digestion procedure [24]. Soil pH and oxidation-reduction potential were measured in a soil-water slurry (1:2.5, w w^–1^) by using an Ultrameter-2 pH meter (Myron L. Company, California, USA). Soil water-holding capacity (WHC, %) and gravimetric moisture content (%) were measured in the laboratory [25].

#### Incubation experiment

The incubation experiment was performed at five temperatures (5, 10, 15, 20, and 25°C) by using three grassland types and six replicates for each grassland type. First, 40 g samples of fresh soil and 10 g quartz sand (preventing soil compaction) were placed in incubation bottles and adjusted to 55% WHC, which is considered optimal for microbial activity. All samples were pre-incubated for 7 days at 15°C to minimize the mineralization pulse [26]. The purpose of pre-incubation was to provide sufficient time for the stabilization of soil microbial populations [27]. A similar experiment showed that the microbial activity in the soils was rapidly restored after pre-incubation [28]. Subsequently, the samples were incubated for 2 days at the five treatment temperatures in different incubators (5, 10, 15, 20, and 25°C) with constant temperature and 80% humidity. We set 56-d incubation experiments to determine the short-term response of *R* to changes in temperature. For these experiments, different incubation temperature were used during the day and night, in order to avoid the temperature-dependence of soil microorganisms as previously reported [29,30]; the samples were incubated at 5, 10, 15, 20, and 25°C during the night and at 8, 13, 18, 23, and 28°C during the day. *R* was measured nine times on days 1, 3, 5, 7, 14, 21, 28, 42, and 56. Soil moisture of the incubated samples was adjusted at 3 d intervals on a weight basis.

#### Determination of SOM decomposition rate (R)

An automatic system for measuring *R* was developed using a modified continuous-gas-flow system [25]. Briefly, *R* was calculated from the slope of CO_2_ concentration and specific transformation factors as follows:

$$R=\frac{C\times V\times \alpha \times \beta }{m}$$ (1)

where *R* is the SOM decomposition rate (μg C g^–1^ d^–1^); *C* is the slope of CO_2_ concentration; *V* is the volume of the incubation bottle and gas tube; *m* is soil dry weight; *α* is the transformation coefficient of CO_2_ mass; and *β* is the transformation coefficient of time [25]. The dynamics of *R* during the incubtaion experiments were provided in S1 data.

The activation energy (*E*_*a*_) of SOM decomposition was calculated using the following Arrhenius equation. The Arrhenius equation was formulated to describe the temperature sensitivity of simple reactions, but can describe the temperature sensitivity of decomposition of complex organic matter well [31]:

$$R=A\times e^{\left(\frac{-E_{\text{a}}}{R_{0}T}\right)}$$ (2)

where *R* is the SOM decomposition rate (μg C g^–1^ d^–1^); *A* is a pre-exponential parameter; *R*_0_is the gas constant (8.314 J mol^–1^); and *T* is temperature in Kelvin (K). By taking the logarithm of both sides of the equation, *E*a was calculated as the slope of the relationship between -1/R_0_T and the natural logarithm of *R*.

The temperature sensitivity (*Q*_10_) of SOM decomposition were calculated as follows:

$$E_{a}=R\times \text{ln}(Q_{10})/\left(1/T_{1}-1/T_{2}\right)$$ (3)

where *Q*_10_ is temperature sensitivity; *T*_1_ and *T*_2_ are temperatures in K and provide the 10-K temperature range for the corresponding *Q*_10_ values (i.e., *T*_2_ = *T*_1_ + 10).

Repeated measure analysis of variance (repeated ANOVA) was used to assess the effects of soil water content, vegetation coverage, vegetation biomass, and soil properties. Univariate analysis was used to test the effects of grassland types (alpine meadow, alpine steppe and alpine desert) and temperature (5, 10, 15, 20, and 25°C) on *R*, the effects of grassland types and temperature range (5–15°C, 10–20°C, 15–25°C) on *Q*_10_, and the effects of temperature range and incubation time (on days 1, 3, 5, 7, 14, 21, 28, 42, and 56) on *Q*_10_. Regression analysis was used to evaluate the relationships between *E*_a_ and *R* at 20°C, as well as the Akaike information criterion. All statistical analyses were performed using SPSS v. 13.0 (SPSS, Chicago, IL, USA), and significance was set at *P* = 0.05.

## Results

### Changes in vegetation community and soil properties

Average coverage of aboveground vegetation was in the following order: alpine meadow (89%) > alpine steppe (20%) > alpine desert (8%). The aboveground biomass was highest in the alpine meadow (147.01 g m^–2^), followed by that in alpine steppe (51.23 g m^–2^) and in alpine desert (14.36 g m^–2^). Coverage and aboveground biomass were significantly different across different grassland types. Furthermore, soil properties differed significantly across different grassland types (*P* < 0.001; Table 2). In the 0–10 cm soil layer, the SOC content was the highest in the alpine meadow (3.62%), followed by that in the alpine steppe (0.74%) and alpine desert (0.16%). Oxidation-reduction potential was in the following order: alpine meadow (166.38 mV) > alpine steppe (137.00 mV) > alpine desert (130.25 mV). Soil pH was the lowest in the alpine meadow (6.58), then in the alpine steppe (8.28) and alpine desert (8.57).

**Table 2:** Soil properties of the experimental plots.

Grassland types	Organic carbon (%)	Microbial biomass carbon (μg g^–1^)	Total nitrogen (%)	Oxidation-reduction potential (mV)	pH
Alpine meadow	3.62 ± 0.58^a^ ^†^	143.97 ± 4.75^a^	0.37 ± 0.15^a^	166.38 ± 8.44^a^	6.58 ± 0.02^c^
Alpine steppe	0.74 ± 0.04^b^	53.02 ± 5.50^b^	0.10 ± 0.01^b^	137.00 ± 1.41^b^	8.28 ± 0.05^b^
Alpine desert	0.16 ± 0.05^c^	27.19 ± 0.29^c^	0.04 ± 0.01^c^	130.25 ± 4.03^b^	8.57 ± 0.04^a^
F	119.028	640.85	16.140	210.985	2929.5
*P*	< 0.001	< 0.001	0.001	< 0.001	<0.001

^**†**^ The values are mean ± 1 standard deviation (n = 4). Data with same letters in the same column indicate no significant difference at the *P* < 0.05 level.

### Changes in SOM decomposition rate (*R*)

**Fig 2:**
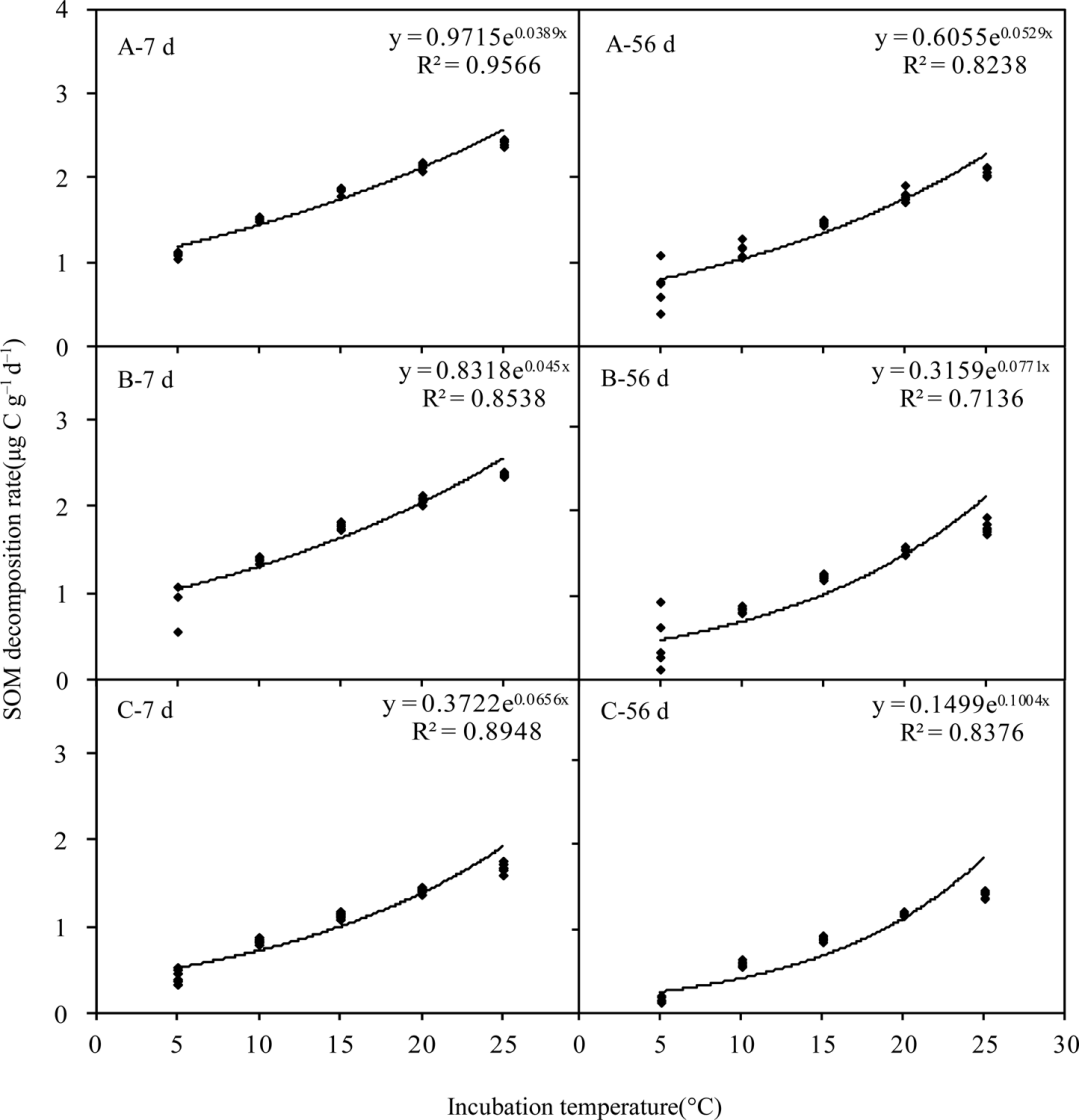
Effects of temperature on the decomposition rates of soil organic matter (SOM) in the different grasslands of the Qinghai-Tibet Plateau. A: Alpine meadow; B: Alpine steppe; C: Alpine desert.

Grassland types had significant effects on *R* (F = 1000.26, *P* < 0.001 for 7 days; F = 239.09, *P* < 0.001 for 56 days; Table 3). Under the same incubation temperature, *R* was in the following order: alpine meadow > alpine steppe > alpine desert (Fig 2). For example, the *R* values at 20°C at 7 days were 2.15, 2.09, and 1.43 μg C g^–1^ d^–1^ for alpine meadow, alpine steppe, and alpine desert, respectively. Moreover, temperature had a significant positive effect on *R* (Table 3), which exponentially increased with increasing incubation temperature (F = 1115.28, *P* < 0.001 for 7 days; F = 440.55, *P* < 0.001 for 56 days).

**Table 3:** Effects of grassland types and temperature on the decomposition rate of soil organic matter (SOM).

	SOM decomposition rate over 7 d		SOM decomposition rate over 56 d	
Source	F	*P*	F	*P*
Grasslands types (G)	1000.262	< 0.001	239.092	< 0.001
Temperature (T)	1115.279	< 0.001	440.550	< 0.001
G × T	1.496	0.173	0.548	0.816

### Changes in temperature sensitivity of SOM decomposition (*Q*_10_)

**Fig 3:**
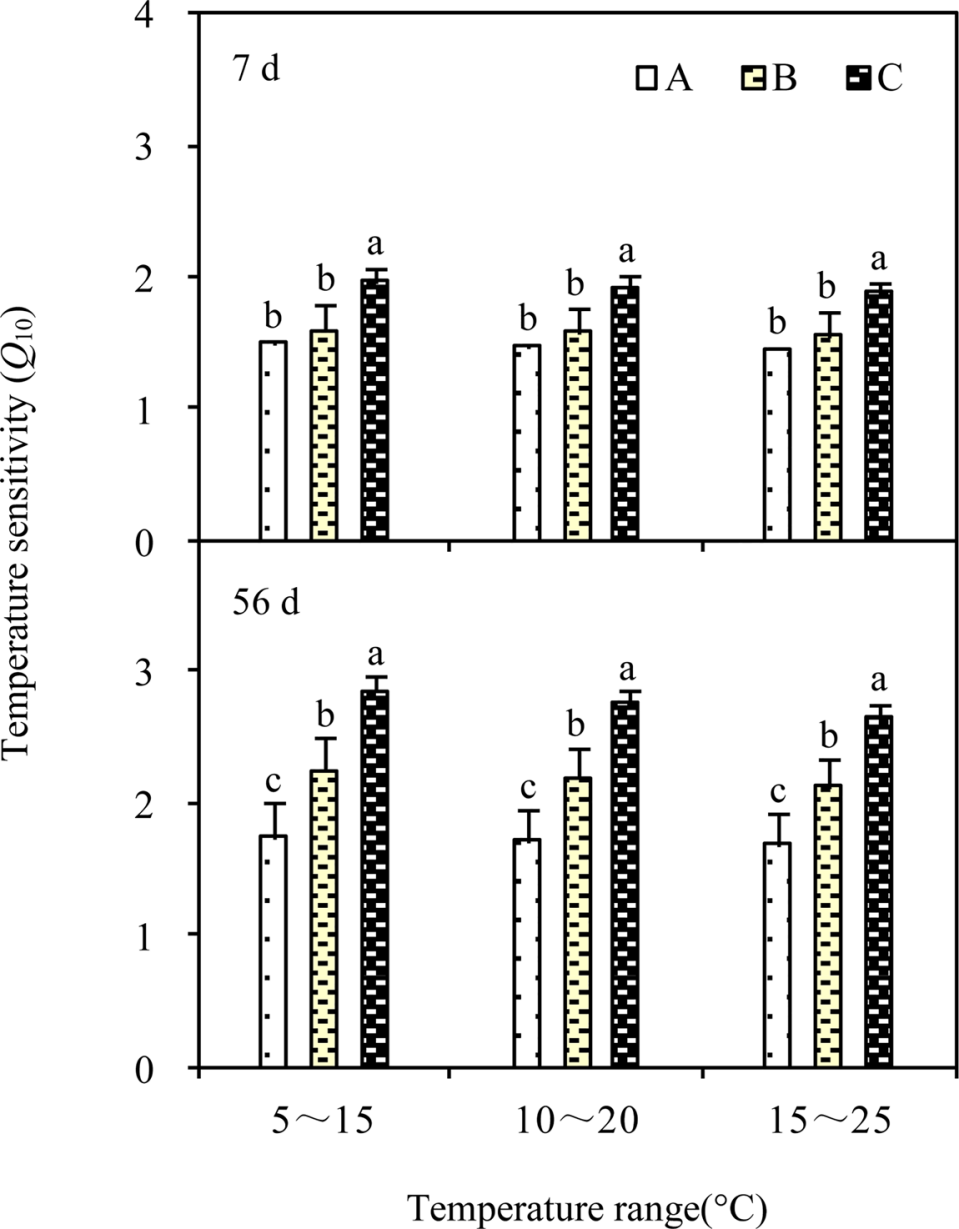
Effects of temperature range on the temperature sensitivity (*Q*_10_) of soil organic matter decomposition in different grasslands of the Qinghai-Tibet Plateau. A: Alpine meadow; B: Alpine steppe; C: Alpine desert. The same small letter in different grassland type indicated no significant difference at *P* = 0.05 level.

The values of *Q*_10_ were significantly different across various grassland types (F = 73.26, *P* < 0.001for 7 days; F = 118.91, *P* < 0.001 for 56 days; Table 4). The average *Q*_10_ values were as follows: alpine meadow (1.56 ± 0.09) < alpine steppe (1.88 ± 0.23) < alpine desert (2.39 ± 0.32); the difference across different grassland types became more obvious in the late phase of incubation (Fig 3). At different temperature ranges, the *Q*_10_ values decreased with increasing temperature range (*P* < 0.05), i.e., *Q*_10_ (5–15°C) > *Q*_10_ (10–20°C) > *Q*_10_ (15–25°C), and varied across different grassland types (*P* < 0.001; Table 4), and incubation times (*P* < 0.001; Table 5).

**Table 4:** Effects of grassland types and temperature range on temperature sensitivity of soil organic matter decomposition.

	Temperature sensitivity (7 d)		Temperature sensitivity (56 d)	
Source	F	*P*	F	*P*
Grassland types (G)	73.258	< 0.001	118.906	< 0.001
Temperature range (T)	1.162	0.322	1.722	0.190
G × T	0.071	0.990	0.157	0.959

**Table 5:** Effects of temperature range and incubation time on the temperature sensitivity of soil organic mater (SOM) decomposition.

	Alpine meadow		Alpine steppe		Alpine desert	
Source	F	*P*	F	*P*	F	*P*
Temperature range (T)	1.877	0.159	0.654	0.522	1.709	0.187
Incubation time (I)	11.475	< 0.001	9.810	< 0.001	15.485	< 0.001

### Changes in the activation energy of SOM decomposition (*E*_a_)

**Fig 4:**
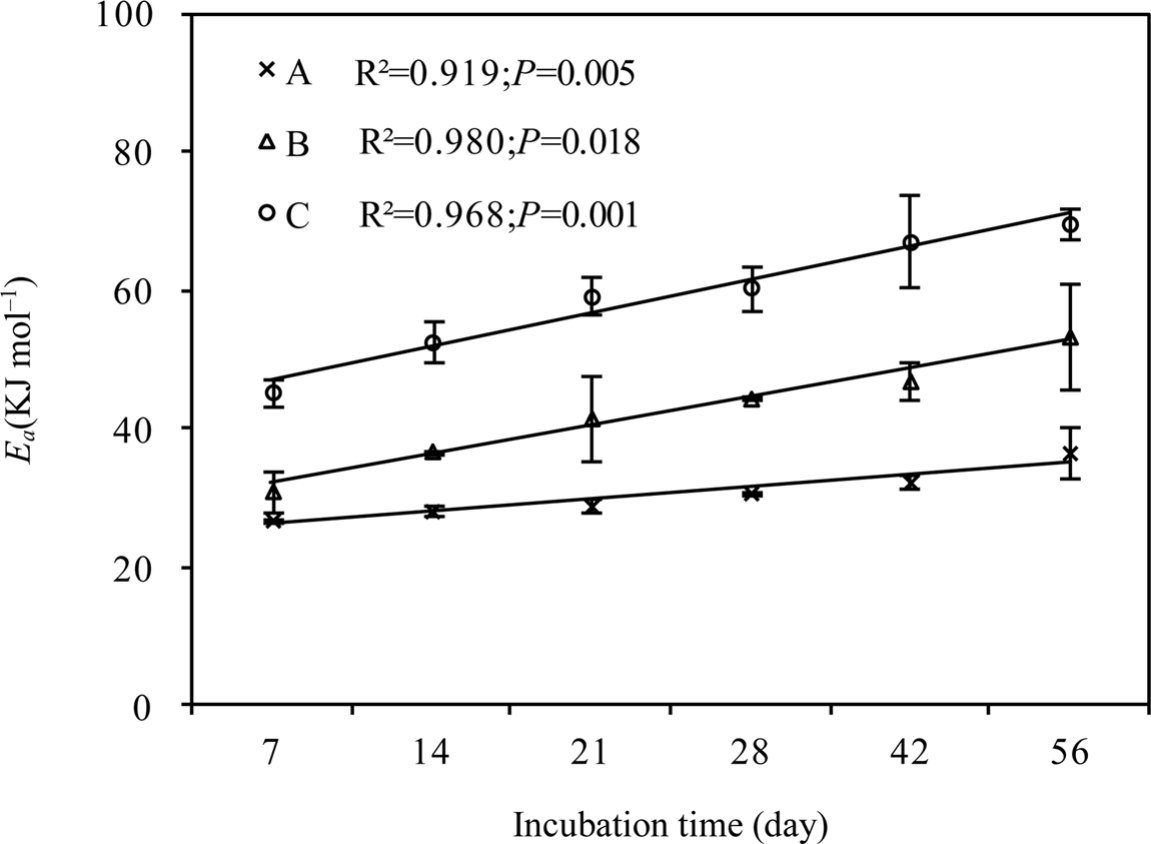
Changes in the activation energy (*E*_*α*_) of soil organic matter decomposition with incubation time in the different grasslands of the Qinghai-Tibet Plateau. A: Alpine meadow; B: Alpine steppe; C Alpine desert.

**Fig 5:**
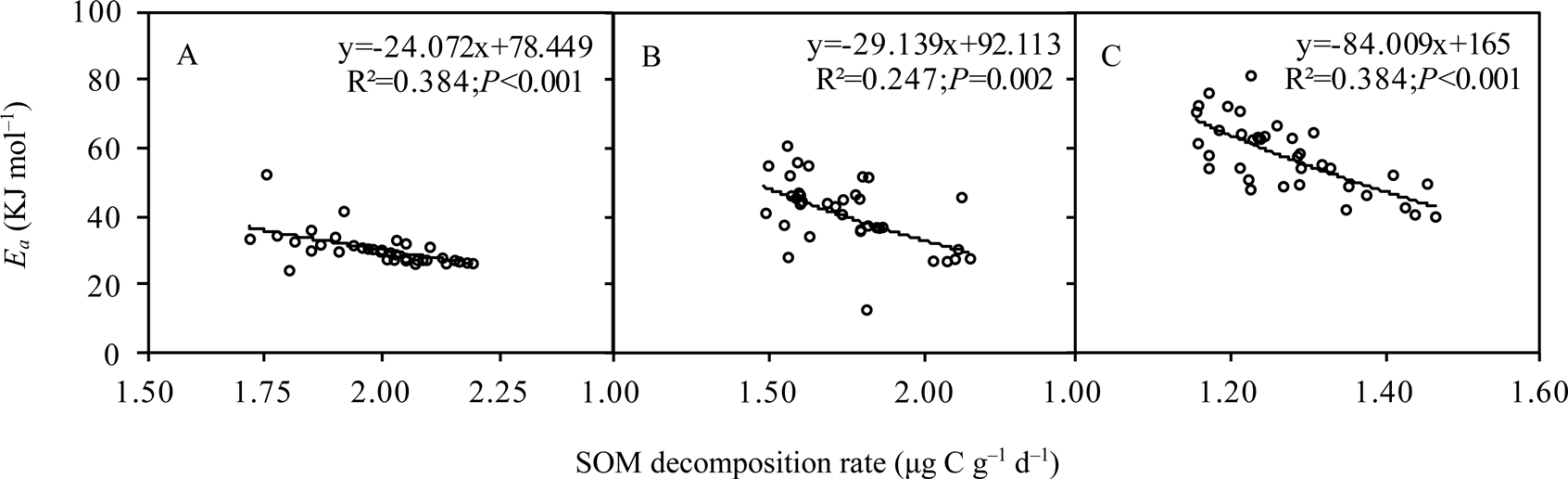
The relationships between activation energy and soil organic matter (SOM) decomposition rate at 20°C in the grasslands of the Qinghai-Tibet Plateau. A: Alpine meadow; B: Alpine steppe; C Alpine desert.

Grassland type (F = 105.043, *P* < 0.001) and incubation time (F = 11.525, *P* < 0.001) had a significant influence on *E*_*a*_. At the same incubation time, *E*_a_ increased in the following order: alpine meadow < alpine steppe < alpine desert (Fig 4). At 56 days of incubation, *E*_a_ was the lowest in alpine meadow (36.61 KJ mol^–1^), followed by that in the alpine steppe (53.44 KJ mol^–1^) and alpine desert (69.73 KJ mol^–1^). With an increase in incubation time, *E*_a_ increased across all grassland types (R^2^ = 0.92, *P* = 0.005 for alpine meadow; R^2^ = 0.98, *P* = 0.018 for alpine steppe; R^2^ = 0.97, *P* = 0.001 for alpine desert; Fig 4). Moreover, across all grassland types, *E*_a_ was exponential negatively correlated with *R* at 20°C (Fig 5).

## Discussion

### SOM decomposition rate differed across grassland types

SOM decomposition rates (*R*) were significantly different across the different grassland types in the Qinghai-Tibet Plateau. This observations indicated that *R* reduced gradually with a decrease in SOC content. The plausible explanation for the differences might be the difference in SOC content (or soil quality), vegetation community, pH, and oxidation-reduction potential across different alpine grasslands [32]. Differences in *R* could be largely explained by the differences in plant biomass, because plant biomass remarkably affected soil physical and chemical properties [33]. At the same time, the differences in aboveground biomass may lead to the alteration of soil microbial community. Changes in microbial community structure or total biomass could induce a measurable variation in SOM decomposition responses to changing temperature [34]. In addition, soil microorganisms release extracellular enzymes into soil matrix to access carbon and nitrogen from the decomposition of SOM. Furthermore, pH is major factors influencing *R* through influencing the availability of carbon and nitrogen for microbial assimilation [35]. During SOM decomposition, microorganisms break down longer carbon chains by oxidation (or open the carbon rings), followed by a complicated biochemical process during which various forms of organic carbons are oxidized to CO_2_, and high redox potential leads to the degradation of the carbon chain, releasing more CO_2_ [36]. Flanagan and Johnson [37] revealed that the differences in *R* were caused by biomass variation in the grassland of North Canada; this could be due to the different basic physical and chemical properties of the grasslands. Similar findings have been drawn in these experiments at landscape scale [31] and at soil depth [38]. In addition, temperature is the main factor affecting SOM decomposition and can be used to explain most variations in *R*. In this study, *R* increased exponentially with increasing temperature across all alpine grasslands, suggesting that temperature increment might promote SOM decomposition in the future to some extent. The response of *R* to temperature changes was closely related to soil microbial activity, and temperature may affect not only enzyme-substrate reaction but also virtually all steps of SOM decomposition, including depolymerization and solubilization of SOM, microbial carbon use efficiency and their enzyme production, as well as adsorption—desorption equilibrium between dissolved SOM and soil solids [39,40,41]. At an optimal temperature range, microbial activity increased with an increase in temperature, leading to an increased *R* [13]. Accompanying with the process of global warming, surface temperatures are anticipated to increase, leading to higher SOM decomposition [4]; the accelerated *R* will increase the quantities of CO_2_ emitted into the atmosphere, thereby exacerbating the greenhouse effect. However, higher availability of soil nitrogen and other nutrients, accompanying with the accelerated SOM decomposition under global warming, might facilitate photosynthesis and growth and enhance the sequestration capacity of atmospheric CO_2_ [42,43]. Therefore, the real effects of warming climate on SOC storage in the alpine grasslands of the Qinghai-Tibet Plateau requre to be investigated further.

### *Q*_*10*_ values differ significantly across various alpine grasslands

The *Q*_10_ values were different across different grasslands in the Qinghai-Tibet Plateau. Moreover, they increased with an increase in incubation time across all grasslands; this indirectly supported the idea that *R* of the alpine desert was more sensitive to temperature changes. Haddix et al. [44] reported that *Q*_10_ varied from 1.3 to 6.5 across six sites in America. Feng and Simpson [45] showed that *Q*_10_ was about 1.2–3.5 in the grasslands of Canada. Ise and Moorcroft [46] reviewed previous findings and found that *Q*_10_ values were 1.4–2.5 at a global scale. Substrate quality is one of the important factors influencing *Q*_10_; these two factors are negatively correlated [47,48]. *Q*_10_ affected by many factors, mainly including the presence of SOM substrates, substrate availability at exo-enzymatic reactionsites, the resource requirements of soil microorganisms, the stoichiometry of SOM compounds, and the *E*_a_ of SOM compounds and thus their complexity [49]. Fierer et al. [50] performed an incubation experiment by using 77 kinds of soil and suggested that there was a significant negative correlation between *Q*_10_ and SOM quality in North America. Consistent with our finding, Hartley and Ineson [13] reported that *Q*_10_ significantly increased with increasing incubation time, indicating that soils with lower quality have higher temperature sensitivity. Therefore, the activity ratio of substrate quality might be the important reason for the difference in *Q*_10_ values across different alpine grasslands.

Some studies have demonstrated that *Q*_10_ values were higher in the regions with low temperature [51,52]. Our findings were consistent with this finding: *Q*_10_ (5–15°C, 2.31) > *Q*_10_ (10–20°C, 2.24) > *Q*_10_ (15–25°C, 2.18). Balser and Wixon [53] reported similar results in North America and assumed that *Q*_10_ was higher at low temperatures in cold areas. Bekku et al. [54] found that *Q*_10_ values were negatively correlated with temperature across different climatic zones. These findings implied that the effect of temperature increment on SOM decomposition was stronger at high latitudes than at low latitudes in the Northern Hemisphere [10]. At this point, this mechanism is only infered from some indirect evidences, and more researches require to determine the prevalence of thermo adaptation by microbial communities and their roles in determining the temperature dependency of SOM decomposition. In future, soil carbon lability, enzyme activity, and microbial community also require to be measured periodically in the process of incubation to explore the underlying mechanims.

### Substrate-quality temperature hypothesis for the Qinghai-Tibet Plateau grasslands

*E*_*a*_ was negatively correlated with *R* in all the three alpine grasslands, indicating that substrate-quality temperature hypothesis is applicable for the Qinghai-Tibet Plateau grasslands. The principles of activation energy suggested that the energy required for SOM decomposition was correlated with substrate quality [55], suggesting that higher *E*_*a*_ associated with the breakdown of recalcitrant substrates result in a greater *Q*_10_ (namely the substrate-quality temperature hypothesis). We tested two corollaries of the substrate-quality temperature hypothesis. First, if biochemical recalcitrance limits the decomposition rate, soil with lower decomposition rates should have higher *E*_*a*_. Second, if the overall lability of the decomposed SOM pool decreases with the prolonged incubation, *E*_*a*_ should increase over time. Several studies have demonstratd how declining responses of SOM decomposition and increasing *E*_*a*_ with increasing temperature can be influenced by decreasing availability of soil substrate [56,57]. Although measuring SOM stoichiometry is feasible, it remains unclear how microbial metabolism with different *E*_a_ vary with temperature [4,58]. A negative relationship between SOM fractions and *E*_a_ can reflect meaningful changes in inherent substrate reactivity over time [59]. It is reasonable to assume that the lower availability of soil substrate results in higher *E*_a_ [49]. Knorr et al. [60] divided SOM into three components and revealed that the *E*_a_ of recalcitrant SOM decomposition was higher than that of labile organic matter. Conant et al. [61] deriveda novel approach by using laboratory incubation (denoted *Q*_10-q_) that accounts for the changes in SOM quality during decomposition (labile *Q*_10-q_ = 2.1 ± 0.2; more resistant *Q*_10-q_ = 3.8 ± 0.3). Furthermore, they [62] found that the *Q*_10_ values of SOM decomposition increased with incubation time. Moreover, the substrate-quality temperature hypothesis predicted that the temperature sensitivity of SOM decomposition increase with increasing *E*_a_ [4]. Further, we generally considered that recalcitrant substrate or low microbialactivity of soil had larger temperature sensitivity of SOM decomposition [61]. To a certain extent, *E*_*a*_ reflected soil substrate and soil microbial activity, and the substrate-quality temperature hypothesis help to refine the models of the soil carbon responses to globalwarming.

## Conclusions

Grassland type and temperature had significant influences on SOM decomposition in the Qinghai-Tibet Plateau grasslands. The *Q*_10_ values were significantly different across different grasslands and were in the following order: alpinemeadow < alpinesteppe < alpine desert, implying the apparent spatial heterogeneity of *Q*_10_ in the different grassland types: more fertile soils had lower *Q*_10_. Moreover, *E*_a_ was exponentially negatively correlated with SOM decomposition rates in all alpine grasslands, and increased with increasing incubation time; both the findings verified the assumption that the substrate-quality temperature hypothesis was applicable to the Qinghai-Tibet Plateau grasslands. This variability should be incorporated into biogeochemicalmodels to better explore the responses of SOM decomposition and storage to warming scenarios in this region.

## Supporting Information

S1 Data Dynamics of soil respiration rates during the incubation experiments. (XLS)
